# A Survey on FPGA-Based Sensor Systems: Towards Intelligent and Reconfigurable Low-Power Sensors for Computer Vision, Control and Signal Processing

**DOI:** 10.3390/s140406247

**Published:** 2014-03-31

**Authors:** Gabriel J. García, Carlos A. Jara, Jorge Pomares, Aiman Alabdo, Lucas M. Poggi, Fernando Torres

**Affiliations:** Department of Physics, System Engineering and Signal Theory, University of Alicante, San Vicente del Raspeig, Alicante 03690, Spain; E-Mails: carlos.jara@ua.es (C.A.J.); jpomares@ua.es (J.P.); aa77@alu.ua.es (A.A.); lmp23@alu.ua.es (L.M.P.); Fernando.Torres@ua.es (F.T.)

**Keywords:** Field Programmable Gate Arrays (FPGAs), rapid prototyping, reconfigurable systems, programmable architectures, low-power sensor systems, smart sensors

## Abstract

The current trend in the evolution of sensor systems seeks ways to provide more accuracy and resolution, while at the same time decreasing the size and power consumption. The use of Field Programmable Gate Arrays (FPGAs) provides specific reprogrammable hardware technology that can be properly exploited to obtain a reconfigurable sensor system. This adaptation capability enables the implementation of complex applications using the partial reconfigurability at a very low-power consumption. For highly demanding tasks FPGAs have been favored due to the high efficiency provided by their architectural flexibility (parallelism, on-chip memory, *etc.*), reconfigurability and superb performance in the development of algorithms. FPGAs have improved the performance of sensor systems and have triggered a clear increase in their use in new fields of application. A new generation of smarter, reconfigurable and lower power consumption sensors is being developed in Spain based on FPGAs. In this paper, a review of these developments is presented, describing as well the FPGA technologies employed by the different research groups and providing an overview of future research within this field.

## 1. Introduction

Processing capabilities in sensor nodes are typically based on Digital Signal Processors (DSPs) or programmable microcontrollers. However, the use of Field Programmable Gate Arrays (FPGAs) provides specific hardware technology, which can also be reprogrammable thus providing a reconfigurable sensor system. The partial reconfiguration is the process of modifying only sections of the logic that is implemented in an FPGA. Thus, the corresponding circuit can be modified to adapt its functionality to perform different tasks. This adaptation capability allows the implementation of complex applications by using the partial reconfigurability with very low power consumption. This last feature also represents an important aspect when FPGAs are applied in sensor systems. Nowadays, the sensor systems are required to provide an increasing accuracy, resolution, and precision while decreasing the size and consumption. Additionally, FPGAs and their partial reconfigurability allow us to provide sensor systems with additional properties such as processing capabilities, interfaces, testing, configuration, *etc.* These sensors are typically referred to as smart sensors.

The current capabilities of FPGA architectures allow not only implementation of simple combinational and sequential circuits, but also the inclusion of high-level soft processors. The use of integrated processors holds many exceptional advantages for the designer, including customization, obsolescence mitigation, component and cost reduction and hardware acceleration. FPGA embedded processors use FPGA logic elements to build internal memory units, data and control busses, internal and external peripheral and memory controllers. Both Xilinx and Altera (the two market leaders in the FPGA industry) provide FPGA devices that embed physical core processors built inside the FPGA chip. This type of processors are called “hard” processors. Such is the case for the PowerPC™ 405 inside Virtex-4 FPGA devices from Xilinx and the ARM922T™ inside Excalibur FPGA devices from Altera. On the other hand “soft” processors are microprocessors whose architecture is fully built using a hardware description language (HDL). The advantage of using such a type of processor is that a designer can implement the exact number of soft processors required by the application offering a large amount of flexibility for the designer. The most famous soft processors are the LEON3 soft processor from Aeroflex Gaisler, which is a Very High Speed Integrated Circuit and HDL (VHDL) model of a 32-bit processor compliant with the SPARC V8 architecture, the Nios II soft processor which is a 32-bit embedded-processor architecture designed specifically for the Altera FPGAs devices and the MicroBlaze soft processor core from Xilinx. This last processor is a 32-bit RISC Harvard architecture soft processor core with a rich instruction set optimized for embedded applications.

Hardware resources that are implemented in FPGAs differ greatly depending on the manufacturer and the specific FPGAs. However, a great number of devices include components that make them adequate to be applied in sensor systems. This is the case of the previous described processors and the implemented transceivers. A transceiver is a serializer/deserializer (SerDes) capable of operating at serial bit rates up to 28.05 Gigabit/second on current FPGAs such as Stratix V FPGA devices from Altera and Virtex^®^-7 HT FPGA devices from Xilinx. They are increasingly used for data communications because they can run over longer distances, use fewer wires, and thus have lower costs than parallel interfaces with equivalent data throughput. Most FPGAs could provide configurable I/O standards in order to allow a wide range of devices to be connected and operated at different voltage levels without the need to use adapter interfaces or voltage converters, significantly simplifying the design and reducing costs. For example, the Spartan-3 FPGA from Xilinx provides various I/O bank standards like LVCMOS, LVTTL, GTL, HSTL, PCI, SSTL, LDT, LVDS, RSDS, and LVPECL that can operate at different voltage levels from 1.2 to 3.3 V.

Some FPGAs incorporate a large amount of arithmetic blocks that can be low-complexity blocks such as simple multipliers or can be relatively more complex like the Digital Signal Processing (DSP) units which consist of combinations of various components like multipliers, adders, accumulators, shift registers, *etc.* A DSP unit significantly accelerates the FPGA's performance and allows achieving greater productivity and flexibility, while decreasing cost and power consumption. For instance, each Stratix II and Stratix II GX device (from Altera) has two to four columns of DSP blocks that efficiently implement multiplication, multiply-accumulate and multiply-add functions. The number of DSP blocks per column and the number of columns available depends on the device, for example, the EP2S180 device from the Stratix II family has 96 DSP Blocks, 769 9 × 9 Multipliers, 384 18 × 18 Multipliers and 96 36 × 36 Multipliers. Furthermore, internal memories offer very high relative speed compared with external memories. Current FPGAs contain large amounts of internal memory blocks, for instance, up to 34 Mb of internal RAM in the Virtex-6 devices from Xilinx. Other memory types can be found such as Random Access Memory (RAM), Read Only Memory (ROM) or shift registers. In addition the designer can implement other memory structures like First In First Out (FIFO).

The cost reduction of the FPGAs, their increasing capabilities and the possibility of improving the performance of sensor systems with specific hardware technologies have led their use in new application fields related with sensors to clearly increase. This paper presents a state of the art overview of the research on sensor systems based on FPGAs in Spain. A great number of applications are integrated in systems that require high data throughputs. Application fields such as image processing and wireless sensors can take advantage of the increasing density of the chips. Nowadays, it is possible to find not only applications in research laboratories, but also in real sensory systems. It is possible to find new fields with growing demand such as thermal management, automotive, robotics, industrial control, medical, reduction of power consumption, *etc.* All these sensor-based applications employ FPGAs with different purposes, as it will be described throughout the paper.

Due to the great amount of data to be processed, computer vision systems currently represent one of the most important fields of research [1–4]. Computer vision systems can be classified into three main levels [5]. The lower level includes the image capture and simple pixel based functions such as arithmetical operations between images. The intermediate level includes operations such as segmentation, matching, preprocessing, convolution or motion estimation. The upper level typically is applied to recognize, classify or capture scene interpretation. Currently, there are a lot of FPGA-based computer vision systems that mainly employ parallel computing in order to increase the processing speed in these computationally intensive applications [6]. High-resolution images and videos require complex compression and coding algorithms [7]. These applications require substantial processing resources and it is possible to find several works that employs the parallel computing features of FPGAs [8–10]. The term Wireless Sensor Networks (WSNs) is currently applied to refer to a set of applications that collect and distribute sensorial data around the sensors [11,12]. Although we can find WSNs with several kinds of sensors, the use of camera as sensor nodes allows the definition of visual sensor nodes that are employed in application such as surveillance. In these applications, the use in the nodes of FPGA-based image processing allows one to satisfy requirements such as low power consumption, small circuitry scale, and reconfigurability of the hardware architecture.

In this paper, a review of the different FPGA-based sensor systems in Spain is presented. Although FPGA research reached a level of maturity in the 1990s, however until the last decade this technology was not implemented in a wide range of applications [13]. Software tools became powerful and hardware resources have been improved in fields such as communications [14], signal processing [15] and cost reduction, which have also played a fundamental role in the development of FPGA technology. Nowadays, the research of FPGA-based sensor systems is well established in Spain and the different research is very heterogeneous. However, as it will be described in Section 2, this research can be classified into different topics. The term smart sensors [16,17] is typically employed to refer to sensors which integrate the use of an FPGA to perform several functions in a single portable device. Smart sensors are devices that are optimally designed to measure specific physical phenomena that are normally difficult to measure. This system is optimal for high-speed applications where online measurements are needed and the reconfigurability feature is required by a specific application. Compression and cryptography is another important research field of interest [18,19]. Sensor data compression is a technique employed to reduce redundancies in order to decrease data storage and reduce communication costs. Another topic within FPGA-based sensor systems is the use of these devices in order to implement acquisition boards [20]. As previously described, one of the more active research fields in Spain is the field of WSNs. These networks are composed of several FPGA-based networks made up of one or several processing elements and sensors. The nodes send the obtained measurements between them or to a gateway. The different components of the WSNs, their nodes and the use of FPGAs to improve the communications and processing aspects are nowadays a promising research field. Another topic of interest is the use of FPGAs for signal processing. The use of FPGAs allows one to acquire and perform real-time processing of the obtained sensor signals. Currently, the variety of the designed FPGA-based controllers is large [13]. Controllers can be found in applications such as robotics, power electronics and motors. Finally, it the FPGA-based computer vision systems that have been mentioned previously can be cited.

A great part of the current research in Spain on FPGA-based sensor systems can be included in one of the topics indicated in the previous paragraph. These topics will be studied in greater detail in Section 2 but, due to the great number of current approaches, a specific section is created to describe computer vision systems (Section 3). In Section 4, the main properties of the FPGA devices employed in the current research are indicated. Finally, the main conclusions that can be extracted from the presented state of the art are discussed in Section 5.

## 2. Sensors Systems Based on FPGAs

This section describes the main research about FPGA-based sensor systems in Spain. These works are classified in the topics shown in the next subsections.

### 2.1. Control Systems

The use of FPGA in industrial control systems is of great interest due to the increasing level of controllers' requirements [13]. The use of FPGAs allows implementing a dedicated parallel architecture that can be adapted to the plant needs in runtime. FPGAs have already been used with success in different sensor control systems, which requires the implementations of fuzzy logic controllers [21,22], motion controllers [23,24], neural network [25–28], control of asynchronous motors [29], power converter controls [30], mechatronic systems [31], *etc*.

The hardware implementation of a control system can improve the speed performance. However, the FPGA resources are limited and the control systems' algorithms must be refined. This last aspect is an important research topic devoted to optimize the FPGA resources in the implementation of control systems algorithms. For example, in [21] a model-based design method for the synthesis of embedded fuzzy controllers for the joint development of hardware and software components is proposed. Although it is possible to implement FPGA sensor-based controllers with floating point arithmetic [32], the required recourses are not optimized with respect to fixed-point calculations. Coordinate Rotation Digital Computer (CORDIC) is a well-known algorithm used to approximate iteratively some transcendental functions by using adders/subtractors and shifters. This approach has been used by several authors in order to refine and optimize a control system to be implemented in an FPGA [33]. Consequently, when control systems must be developed in an FPGA, a compromise between control performance and complexity of the hardware architecture must be achieved. In the next three subsections, the main FPGA-based controller applications are classified in image-based controllers, advanced control approaches and monitoring systems.

#### 2.1.1. Image-Based Controllers

As previously described, image information can take advantage of the parallel processing capabilities on FPGAs [4]. This information provides global information about the workspace and is progressively integrated in the control systems. In [34], a neuro-inspired mobile robot with a double spike-based control mechanism for two DC motors is proposed. All the image processing issues are also carried out in an FPGA (capture, processing and line tracking). A similar approach is presented in [35] where an address-event representation is employed for visual sensing, processing and finally actuating a robot. In [36], a hardware/software design and implementation for localization of robot in Mars rover missions is presented. This last paper proposes a system architecture implemented on a Xilinx Virtex-6 FPGA to process the obtained images, perform the visual slam, 3D map reconstruction and to obtain the location of the rover at the map. In [37], a high precision automatic system for liquid level measurement in membrane distillation applications is presented. This approach is based on the laser triangulation principle using two lasers and a camera. The level measurement is obtained by an FPGA that performs the image processing. In [38,39] the Simple Network Robot Protocol (SNRP), which permits the integration of network robots and sensors, is defined. In this case, an FPGA has been used to implement a real-time vision system that provides SNRP services to the network. Using the FPGA computer vision module and the SNRP protocol it is possible to implement visual servoing algorithms for industrial robots.

#### 2.1.2. FPGA in Advanced Control

Currently, FPGAs are being applied to implement not only classical control systems, but also different kind of control systems such as predictive control, fuzzy systems or neural networks. Predictive control is a well-established control strategy that is being used in an increasing set of application areas. The parallel nature of these controllers fits well with the architecture of the FPGAs [40] and different implementations can be found in the literature to operate on any variable (temperature, speed, pressure, *etc.*). In [30] the use of these controllers and their optimal implementation on an FPGA for application in power converters are described. Another kind of control system that is now implemented with success in FPGAs, are the fuzzy controllers [22]. These systems do not require complex modeling of the plant and the control strategy is defined by using linguistic rules that can be implemented using an FPGA architecture. The high computational load of the fuzzy algorithms can be processed using the parallel FPGA architecture to achieve the desired accuracy in real-time. In [21], a model-based approach to implement fuzzy controllers on an FPGA is proposed. Other specific implementations of these controllers can be found in applications such as automotives [41] or education [42]. The increasing capabilities of the FPGAs have opened a new line of research investigating methodologies for scaling intelligent controllers or architectures into embedded systems based on FPGAs [43]. This can be accomplished by the implementation of neural networks [44]. For example, a neuro-inspired mobile robot controller with two DC motors has been proposed in [34]. An important research line is to optimize neuro-inspired models that simulate the neuron layers in the brain. In [3,24,45] a spike-based Proportional-Integral-Derivative (PID) controller is proposed based on FPGAs. The spike-based codification mimics the neuron functioning. The spiking neurons are excited by streams of pulses (spikes), and their output is just another stream of spikes. In the previous references, this information is employed for process visual information and tracking.

#### 2.1.3. Monitoring Systems and Control

The use of FPGA also allows the reduction of delays in the control system feedback. Highly demanding data throughputs can take advantage of the ever-increasing density of the chips in FPGAs [46]. Several applications require not only the capture of sensor information in the feedback but also to process such information in order to obtain the required data to be compared with the system reference. Within this topic, one can mention the work described in [47] where a monitoring infrastructure based on FPGA is proposed. In [37], a computer vision system is presented for liquid level measurement in membrane distillation applications. Another monitoring system is presented in [48–50]. In this case, thermal sensors are employed and they can be used to detect, for example, if a given device dissipates excessive power or does not work correctly.

### 2.2. Smart Sensors

The demand for small sized, high accuracy and low consumption smart sensors has grown over time. The term smart sensor is frequently employed for sensors that integrate several functions in a single portable device such as communications capability, self-diagnostics, decision-making and some “intelligence”. Therefore, the different topics described throughout this paper can be considered as part of a smart sensor: network sensors, control, signal processing, *etc.* These options are commonly integrated in an embedded FPGA-based device when the term smart sensor is employed. The use of FPGAs and their reconfigurability feature allows the addition of different capabilities such as signal conditioning and signal processing [16,51,52]. Furthermore, a smart sensor not only provides the sensory information but also performs additional functions for error compensation or for obtaining complex data from that measurement (see e.g., [53] where resistance and capacitance information is extracted from the sensor data or [48,49] where FPGAs are employed to include additional features to thermal sensors [54]).

The term smart camera is currently employed for cameras that combine video sensing, processing, and communication on a single embedded platform [17]. The integration of the hardware and software components of a computer vision system in a single portable smart camera is a challenging task. The capacity of the FPGAs to process large image data has allowed the integration of low and mid-level vision algorithms in an embedded smart camera [55]. In this case, the camera does not provide an image but processes data from the image. This approach is optimal for high-speed applications or those that requires the processing of a large amount of data such as tactile information [56].

### 2.3. Sensor Networks

A sensor network consists of a set of autonomous devices (sensor nodes) connected to a network and distributed in an area susceptible of study. These devices use sensors to monitor physical or environmental conditions, having restrictions on computing power, communication and energy concerns. The term WSNs, already defined in the Introduction section, refers to a sensor network that employs wireless communication. Currently, the number of applications for WSNs has grown hugely in several areas (automation, image processing, security, telemedicine, robotics, domotics, *etc.*) [11]. The main feature demanded for these applications is reduction of the power consumption because the nodes are usually low-cost sensors operating in an environment with limited processing power and restricted battery autonomy. Therefore, low energy WSNs are needed in engineering fields in order to get the longest lifetime possible. For that end, dynamic reconfigurable devices such as FPGAs allow important improvements concerning energy efficiency, because of their efficient use of the communication channels. Moreover, in this case the FPGAs work as distributed reconfigurable devices that permit the implementation of different functionalities everywhere using remote resources. Most of the contributions in the scientific world try to make the most of the FPGAs in order to reduce the transmission of data among the sensor nodes [57,58], to change dynamically the frequency [59,60] and to turn on the radio transceiver selectively [61]. This subsection describes the main approaches developed in Spain concerning the use of FPGAs in sensor networks, where it will be seen that they are related with the purposes above commented.

In [62], a distributed architecture for integrating micro-electromechanical systems was presented. Each micro-electromechanical system is connected to a smart sensor implemented in an FPGA. The FPGA implementation performs the functions of signal conditioner and communication interface, making the designed nodes small in size, flexible, customizable and reconfigurable. The distributed architecture uses the time-triggered master-slave protocol, where both the master and slave nodes have been developed with the same kind of FPGA. A TX/RX unit, a buffer tri-state to access to the bus, a master controller with the time-triggered protocol integrated and a dual-port memory to supply the information related to the system connected to the network, are used within the master FPGA. Similar components are employed in the slave FPGA with an additional hardware divisor in order to obtain the transmission rate.

As stated, FPGAs allow important improvements concerning energy efficiency because of their efficient use of communication channels. A recent idea to improve power consumption in WSN applications is to be able to switch off the main components of a sensor node. Therefore, the hardware device is only activated to accomplish a given task when it is externally demanded. For that purpose, a low power radio that remains always active is used to activate some needed components of the sensor node. This is known as Wake-up Radio (WuR) and it is employed in on-demand WSNs. This idea has been implemented in [63], where FPGAs are used to implement WuRs for WSNs in order to improve the energy efficiency of the task over a traditional micro-controller architecture.

An important contribution in the field of heterogeneous WSNs is the R-GRID platform [64]. This platform consists of a set of distributed reconfigurable resources that can be integrated according to the grid model. The R-GRID approach was developed to facilitate the implementation of multiple-users and multiple-application-instances, dealing with the complexity of distributed heterogeneous FPGAs. For that end, R-GRID uses virtualization techniques to decouple the behavior of the hardware resource from the physical implementation level. In addition, the approach presented in [65] describes the implementation of a fast decision algorithm for the connectivity of mobile sensors with heterogeneous wireless networks using FPGAs. The FPGA device is installed and embedded in the mobile terminals and adjusts a set of weights to improve the Quality of Service (QoS). Furthermore, another multi-purpose sensor network approach using FPGAs is proposed in [47] for delay-based measurements.

The hardware design of the nodes is critical for WSNs in order to be adapted to the application requirements. A modular sensor node can adapt the hardware platform to different scenarios allowing rapid prototyping and low redesign effort. For that end, dynamic reconfigurable devices such as FPGAs play an important role because they can add flexibility to the sensor node. In this context, an important approach developed in Spain is the Cookie platform [4,19,66,67], a modular device that has an innovative WSN node architecture. This modular platform is divided into four functional layers: communication, processing, power supply and sensing/actuating layer. The heart of this platform, the processing layer, includes a microcontroller and an FPGA device, giving more processing power and flexibility to the platform. This layer carries out the processing of all the information given by the sensors. On the one hand, the microcontroller usually deals with the communication control. On the other hand, the FPGA processes the signals coming from sensors. This platform has been tested in processing, power consumption, communication, and encryption with successful results [19,67].

Another relevant issue for WSNs is to compute the location of the mobile nodes connected to the network. For outdoors, location technologies such as GPS or Galileo can be used. However, for indoor environments, technologies based on RFID, image recognition or ultrasonic must be employed. In this context, in [68], a low cost ultrasonic-based location system for mobile nodes is presented using FPGA devices. This location system is employed to obtain the maximum reachable precision. On the one hand, an FPGA device is used to excite the ultrasonic transmitter, and on the other hand, another FPGA device is employed in the mobile node to identify the time difference between the obtained measurements.

### 2.4. Signal Processing

Embedded signal processing is another topic of interest in the use of FPGAs. Until the appearance of FPGAs in the electronic world, DSPs were the key devices for signal processing. Currently, for highly demanding tasks, FPGAs have superseded DSPs due to the high efficiency given by their architectural flexibility (parallelism, on-chip memory, *etc.*) [69], reconfigurability [70] and massive performance in the development of algorithms [71]. This subsection provides a brief explanation about the main Spanish approaches in the use of FPGAs for signal processing in sensor systems.

In most cases, FPGAs are used for the implementation of sensor data processing. In this context, in [14], the design of WSNs to get the data of a set of pulse oximeters is presented. In this paper, pulse and oxygen values are processed in the FPGA and the obtained values are sent in real time to the Database Server via a WSN. Another contribution to mention is the presented in [72], where some spike-based band-pass filters have been synthesized for FPGA devices.

Low-level processing of ultrasonic signals is another issue which is being implemented with FPGA devices in order to increase scan rate, precision, and reliability [15,20,73,74]. In this context, using Time-Of-Flight (TOF) measurements given by the transducers, some drawbacks such as cross-talk problems, specular reflection and echo discrimination can arise and generate errors in the distance computation. In order to solve these problems, multimode techniques such as Golay sequences [15] are employed. The implementation of this algorithm in an FPGA device permits adaptation to the distance of the reflector in the environment, simultaneous emissions and simultaneous reception in all transducers being able to discriminate the emitter of the echo.

### 2.5. Other Sensors

Data compression is a technique that often improves the data bandwidth requirement of any sensor system. FPGAs can be used to implement different kinds of data compression [7,75]. The design of a compression scheme depends, obviously, on the application. In [18,76] a compression scheme over an FPGA is described. The works presented in these papers can be applied to any multi-sensor that must send and receive data simultaneously from different independent sources. Parallelism properties of FPGAs schemes are exploited to implement different units in charge of each of the signals emitted. Sending more than a data source at the same time is the main idea of these papers. This idea was firstly developed by Hernandez *et al.* in [74], where an ultrasonic sensor is improved by sending data simultaneously. Using both, an FPGA and a DSP, the system is able to receive and process this data in real-time. FPGAs can also contribute to decide which kind of image compression fits better with a given image, as in [77], where the authors apply their compression selector in an FPGA embedded on a satellite. An FPGA is a device that really improves the behavior of parallel algorithms. There are also applications exploiting this parallelism like the FPGA-based web servers shown in [78].

Another application where an FPGA may help is in reconfigurable data acquisition systems [79]. Data acquisition systems are used in vast range of tasks. In [80], an FPGA is used as a thermal sensor to measure the behavior of ring oscillators over different voltages. This sensor measures whether a given device dissipates excessive power in relation to the input voltage. The program is implemented through a Microblaze microprocessor over a Xilinx FPGA. Another example of reconfigurable data acquisition system developed over an FPGA is found in [81]. Errors and interference caused by long wires in certain sensors can be easily solved by adding embedded processing units to the sensor. The case of piezoresistive tactile sensors is worked out in this way by the authors. They come up with the implementation of processing units using standard microcontrollers, Programmable Systems on Chip (PSoCs) and FPGA. The results show that the performance of FPGA solution is closer to that of Application Specific Integrated Circuits (ASICs) rather than that of the other devices.

## 3. Computer Vision Systems Based on FPGAs

Computer vision systems are traditionally based on a sequential architecture. Thus, image processes are run one after another in succession. The program is divided into a sequence of arithmetic and logic operations that are performed by the Arithmetic Logic Unit (ALU). The rest of the CPU is designed to supply the ALU the required data. The algorithm is compiled into a sequence of instructions, which are used to control the operations performed by the CPU and ALU per clock cycle. Therefore, the basic operation performed by the CPU is to seek an instruction in the memory, decode it to determine which operation should perform and execute it.

In contrast, a parallel approach implements any instruction from any algorithm on a separate processor. Nevertheless, if the algorithm were predominantly sequential, with each of its instructions depending on the data from the previous instruction, the gain that could be obtained would be practically zero. In order to get a useful parallel implementation, the algorithm must be susceptible to be split into independent parts and to achieve any significant gain, the portion of the algorithm that can be implemented in parallel must be significant. Luckily, the image processing is inherently parallel, especially in the mid-level and low-level tasks. The complexity in algorithm translation from sequential to parallel opens a very interesting research topic that is reviewed in this section. Works like [82–84] describe some technique to minimize the time required to perform a computer vision algorithm on an FPGA.

**Figure 1.**
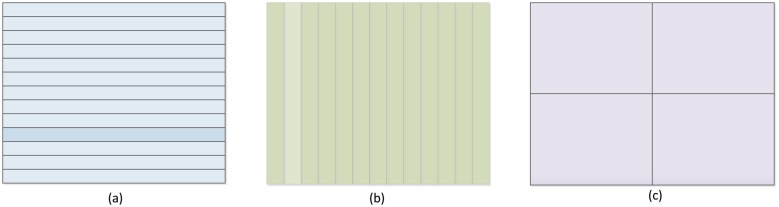
(**a**) Row partitioning; (**b**) Column partitioning; (**c**) Block partitioning.

Two different parallelism concepts can be applied to computer vision algorithms: temporary parallelism and spatial parallelism. Image processing algorithms generally implement a sequence of image operations. By assigning each procedure to a different processor, image processing can achieve a temporary fashion of parallelism. This would be a pipelined architecture, where each processor carries out an operation on the data and sends its results to the next entity. Image processing algorithms often contain one or several loops. These loops iterate over all pixels of the image, applying the same operation, regardless of its value. This kind of parallelism is called spatial parallelism. In order to take advantage of spatial parallelism, the image must be partitioned in some fashion (see Figure 1). After this, a different processor can process each part of the image.

**Figure 2.**
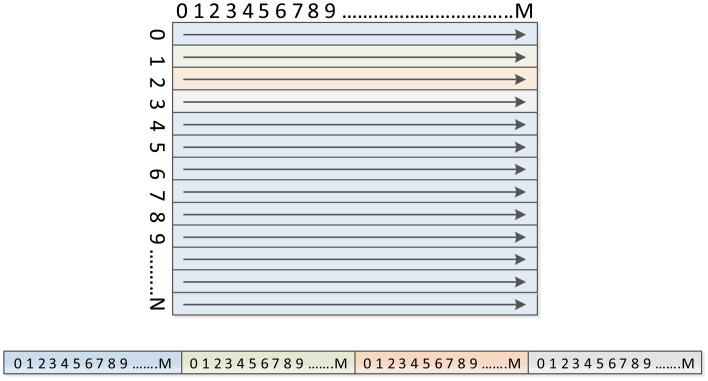
Image stream.

The necessary time (and bandwidth) to read the image from the memory and to store it after being processed is one of the most common bottlenecks in image processing. Converting the spatial parallelism into a temporal parallelism can help minimize this problem. To do so, the image is streamed, *i.e.*, the image is read and written sequentially using a browser frame, usually at a speed of one pixel per clock cycle (see Figure 2). From this image stream, the time spent on the image processing is obtained from the amount of time required to read/write the image and the processing latency. In most operations, latency usually takes much less time than the loading of the whole image itself. So if the algorithm of image processing can be implemented in a flow, the response time will be dominated by the frequency at which the images are provided.

In order to describe the task that a computer vision system must develop, three abstraction levels have been widely assumed in the literature: low-level, mid-level and high-level tasks [5]. Low-level vision tasks consist of pixel-based operations such as filtering, intensity estimation, segmentation and edge detection. In a low-level task, the computer vision system usually deals with a large amount of data. These tasks consist of small neighborhood operations such as segmentation, filtering or basic point operations. However, low-level tasks require, generally, relatively simple operations (such as multiply and add). Mid-level vision consists of pixel grouping operations such as determining object features or region labeling. These tasks are again characterized by local data access, but more complex pixel operations. Finally, high-level vision tasks are more decision-oriented, such as object recognition, face recognition or scene recognition. These tasks involve non-local data access and non-deterministic and complex algorithms. The same task can often be referred as any of the three levels in the literature. However, in this paper the different works about vision and FPGA have been divided into these three categories following the next rules: if the primary purpose task is image enhancement, the task is categorized as low-level; the tasks that operate on the pixels to produce features in the image are mid-level tasks; and finally, decision-making stage is classified as a high-level task.

### 3.1. Low-Level Vision Tasks

FPGAs are ideal for image processing, particularly for low-level and mid-level tasks where parallelism is exploited [9]. Most of the works found in the literature related to computer vision and FPGAs describe a parallelism version of a classical sequential computer vision algorithm [9,10]. For a pipelined architecture, a different hardware block is built for each image processing operation. The block implementing the image processing operation passes its processed data to the next block, which performs a different operation. When the system is not synchronous, intermediate buffers between operations are required. These buffers handle the variations in the data flow. As stated before, building multiple copies of implemented operations and assigning different partitions of the image to each copy can exploit spatial parallelism. A full spatial parallelism can be achieved by building a processor for each pixel. In practice, high image resolution of modern cameras makes this unlikely.

Logical parallelism is the overall parallelism contained in a program, *i.e.*, all the computations that may, according to the semantics of the programming language, be executed in parallel. The logical parallelism within an image processing operation fits into an implementation on the FPGA. This is where most of the image processing algorithms can significantly improve performance. To do so, inner loops are unrolled. Thus, operations are performed in parallel hardware instead of sequentially.

**Figure 3.**
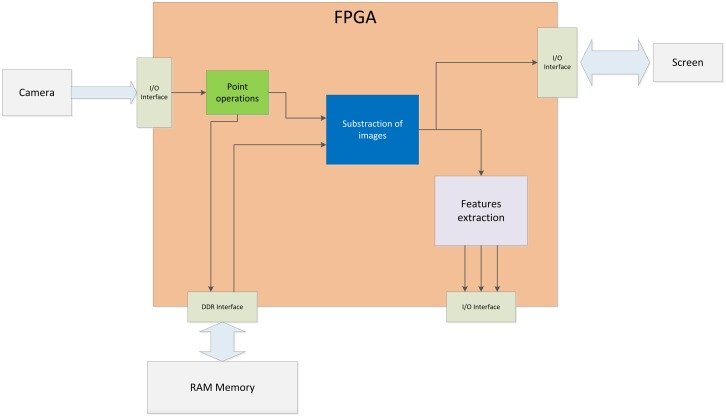
Scheme of a computer vision system embedded on an FPGA. Four points are tracked, and their center of gravity computed at camera frame rate frequency.

Figure 3 depicts a scheme of a low-level to mid-level vision task implemented over an FPGA. Parallel skills have effects on the construction of the vision system [9]. Implementing a pipelined architecture in an FPGA permits operating at the same frequency pixels are served. Given that power consumption is directly related to the clock frequency, a lower frequency implies a lower power demand by the system. The vision task described in Figure 3 is a typical FPGA approximation to an image processing task.

**Figure 4.**
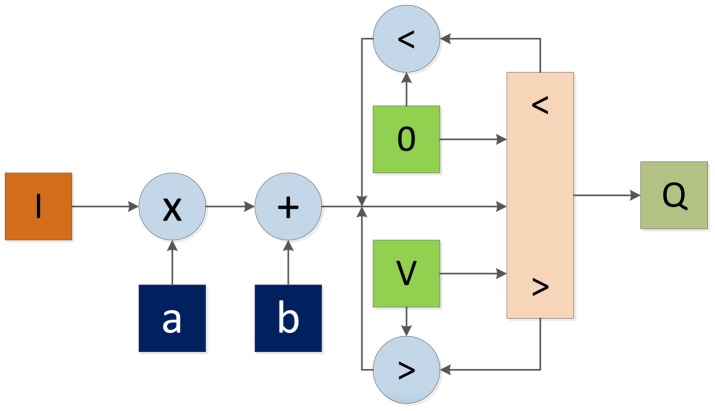
Basic point operation on an FPGA. Input pixel luminance value is multiplied by the constant *a*, and then summed with the constant *b*. The value obtained is clipped before storing the new Q value of the pixel.

Normally, the image data goes serially, which fits perfectly in a hardware implementation, especially if it is possible to interface directly to the camera. Anyway, a block (represented in Figure 3 as an I/O interface directly connected to the camera) performs the communication with the camera to receive the flow of pixels from the sensor. This block is responsible for implementing the required protocol (I2C, Camera link, *etc.*) to communicate with the capture device, configure it and get the image stream. Once configured and initiated the transmission of data, the flow of pixels is driven into the basic image processing block (Point operations green block in Figure 3). This block represents a low-level vision task block. Point operations have widely used in terms of contrast enhancement, segmentation, color filtering, change detection, masking and many other applications. These operations contain the peculiarity that the output pixel depends only on the value of the input's pixel. Figure 4 depicts an example of this kind of module, where a simple contrast enhancement operation to the input image is performed. The constants *a* and *b* with two simple math operations over input pixel value provide a new luminance value. This value may exceed the range of representable values. Thus, the result must be clipped. In Figure 4, this clipping operation is performed over the output value. Operating with the input value may improve the performance in a parallel scheme because both, math operations and logic comparisons can be processed concurrently with two processors. The result of this module can be stored in some kind of device memory (DDR2 RAM in Figure 3). This last step is not strictly necessary. A buffer storage is required only for system synchronization.

**Figure 5.**
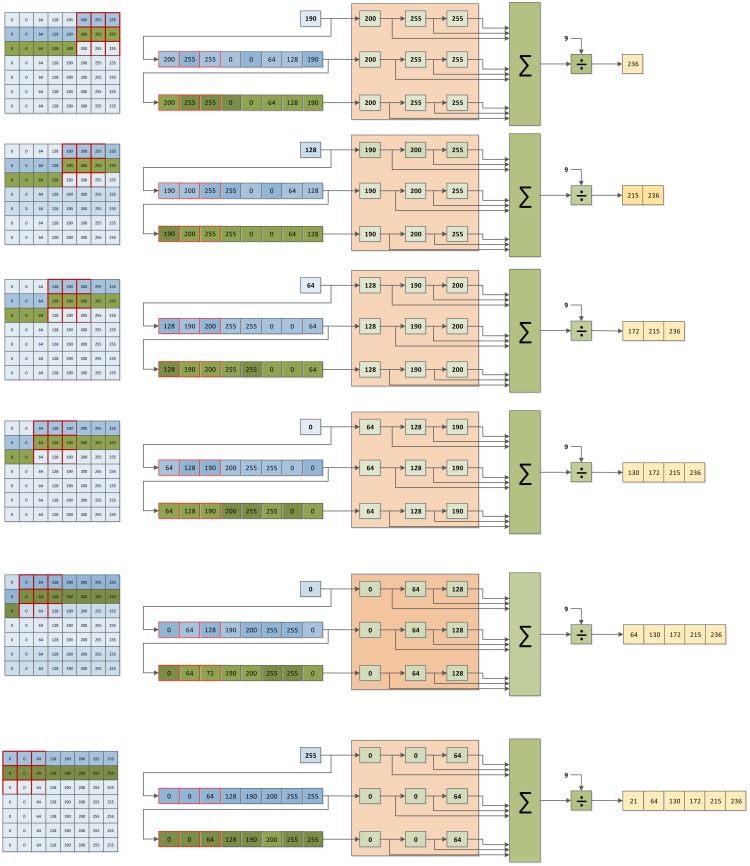
Image average filter on an FPGA. Different successive iterations of the image processing operation.

Point operations are just the basic low-level vision tasks. Normally, from the enhanced image obtained by a point operation module, the computer vision system performs other low-level operations like an image average filter. Filters or blob tracking operations have in common that they need more information besides the value of the pixel being processed. To do so, providing with the necessary architecture to obtain such information (vector structures, intermediate buffers, *etc.*) is essential. Figure 5 shows some iterations of an image filter computed in an FPGA. On the left the input image is represented for each iteration. The red grid remarks the convolution mask employed to compute the central point in the correspondent iteration, whereas row buffers are depicted in a darker blue and green. Row buffers values are also shown in the right scheme of each iteration. The window mask buffers are represented in orange. Row buffers and window buffers are updated by iterating over the image stream. Parallelism is exploited thanks to these buffers. From window buffer, the simple average can be computed at each iteration. The outcome is a valid pixel value of the output filtered image.

One of the basic low-level vision tasks is an image convolution. This was also one of the first processing image issues to be implemented in an FPGA [6,33,85]. In this work, images provided by a high-resolution sensor were passed to the FPGA. Then, the program embedded on the FPGA applied a convolution with a mask over the image and afterwards transmitted that preprocessed image to a PC. Recently, this basic operation was used to obtain object's edges of an image provided by a spiking system [86]. A spike system also called Address-Event-Representation systems (AER) is a camera sensor that computes internally the movement of the objects in the scene. When a pixel changes its luminance, an event is generated and this is the information transmitted by the camera to the computer vision system. In this paper, Linares-Barranco *et al.* present two FPGA implementations of AER-based convolution processors. In [87] a design based of FPGA device is described, used in spiking systems for real time image processing. In this case, the AER device described is a synthetic AER retina emulator, used to simulate spiking retina behavior getting as video source a standard video composite source. This design has been synthesized into synchronous and asynchronous FPGA devices to compare their capabilities. Another project related to AER sensors that uses an FPGA is the described in [45]. In this project, the FPGA can perform five different functions: turn a sequence of frames into AER in real time; histogram AER events into sequences of frames in real time; remap addresses using lookup tables; capture and time-stamp events for offline analysis; and reproduce time-stamped sequences of events in real time. In [88] an FPGA is used to develop a real-time high-definition Bayer to RGB converter. Two image processing operations were parallelized in order to obtain this converter: bilinear interpolation and a new median filter scheme that does not require extra memory and is able to work in real time.

Motion estimation represents a highly descriptive visual cue that can be used for applications such as time interpolation of image sequences, video compression, segmentation from motion or tracking. Optical-flow algorithms have been widely employed for motion estimation using FPGAs [32,89–91]. Different approaches to the subject include image block-matching, gradient constraints, phase conservation, and energy models. In [55] Botella *et al.* present a work developed over a Xilinx board that performs two low-level vision tasks: gradient family optical flow estimation and variant orthogonal moments. These two blocks are then used for a mid-level task (tracking). The system described in [2,32] shows how an optical flow estimation circuit can be implemented using an FPGA platform to achieve real-time computation. The difference in this proposal lies in the fact that authors implement a classical gradient Lucas and Kanade model [92]. They compare different optical flow estimation methods to evaluate the performance of the system.

Adaptive fovea imagers define non-concentric reconfigurable structures for rectangular fields of view. Following procedures used in vision pyramids, from the uniform resolution images supplied by the camera, the upper levels are computed progressively reducing resolution and data volume. In [93,94] adaptive fovea imagers are implemented in an FPGA. Each pixel of the full resolution image is averaged in a low-level vision task. Another interesting image processing application where an FPGA increased the performance is in on-line fingerprint matching [95]. In [96] the FPGA implementation of the structural analysis algorithm consists of a finite state machine core block responsible for managing the neighbourhood analysis. In order to accelerate the computation of distances and angles among minutia points a CORDIC coprocessor is implemented. CORDIC is commonly used when no hardware multiplier is available since the only operations it requires are addition, subtraction, bitshift and table lookup [97].

Stereo correspondence is a low-level vision task. It was not always considered in this group. Nevertheless, no high level information helps the viewer in matching points obtained from the two images of a stereo pair. Stereo vision is a particularly interesting sensor for space vehicles like rovers. Thus, stereo vision matching for embedded systems like FPGAs has been widely researched in the literature [89,98,99]. Stereo matching algorithms can be classified into two approaches (global and local) based on the strategies used for estimation. Global approaches result in more accurate results but at a higher computational cost. In [100] Barranco *et al.* implement two different alternatives to compute the vector disparity for an active vision system: a gradient-based technique, the local algorithm of Lucas and Kanade and a phase-based one detailed in [101] (also a local algorithm). The first technique estimates small local disparities assuming the intensity or brightness constancy of a pixel between left and right images, while the second one computes the disparity using the phase information for different orientations, in a contrast-independent way. Both methods have been implemented over a Xilinx Virtex4 XC4vfx100 device, and they achieved a working frame rate of 32 fps with a 640 × 480 resolution. Their tests validate the proposal and conclude that Lucas and Kanade algorithm is the best choice. Gil *et al.* [102] describe a mobile robot guiding application, based on an FPGA, which computes stereo correspondence on a pair of images coming from a stereo rig. The stereo algorithm implemented is based on the Census transform, described in [103]. In [104,105] one of the most used global algorithms is implemented in an FPGA: belief propagation. It is a matching algorithm of high precision, but it requires a lot of memory. This memory requirement is even worse for high definition images. The architecture proposed uses a Xilinx Virtex 5 330 VLX FPGA to reduce the execution time required to obtain high definition depth maps.

It is common to find in the literature works that implement only part of the system over an FPGA and the rest is implemented in a PC or another system. Normally, processing image is embebbed on the FPGA, whereas decision tasks (correspondent to high-level vision tasks) are developed over a conventional processor. In [37] the FPGA board allows a concurrent operation as the acquisition of the image and the processing of the data can be performed simultaneously. The processing block includes image filtering, threshold level adjustment and edge detection. The image processed is then passed to a computer in order to measure the high-resolution simultaneous dual liquid level in membrane distillation application.

### 3.2. Mid-Level Vision Tasks

Normally, the input for a mid-level algorithm is an image processed in a low-level task. Information delivered at this stage corresponds to features of the image itself or of the objects contained in the image. Examples of these are estimation of blobs position, magnification, orientation, corner or edge detection [8], or region labeling.

The great majority of the works including an FPGA have been conducted to obtain a smarter camera sensor. In [17] a binary discrete time Cellular Nonlinear Network (CNN) camera prototype based on an Actel IGLOO FPGA is proposed. The camera is employed to guide a LEGO Mindstorm robot. Embedded sensor must fit the LEGO Mindstorms electronic sensors requirements in terms of device size and power consumption (less than 140 mA). The low-power consumption of the FPGA, and its reconfigurability are exploited in this work to perform corner detection. The work presented in [1] describes the use of an FPGA to compute the relative pose of an underwater robot with reference to a pipe. After an image binarization, FPGA computes the distance between the lines of the pipe appearing in the binary image, the position of the center of the first line and detects if one of the lines has disappeared from the image. From this data a DSP computes parameters like angular displacement and the distance between the robot and the pipe. Local low and mid-level processing ends here as this information is sent to a host, which is in charge of the final steps concerning scene understanding and interpretation tasks (high-level vision tasks). Blob moving detection over a static background is one of the most common tasks undertaken in computer vision. Navigation, tracking and surveillance applications are directly involved with movement analysis. In [106,107], Principal Component Analysis (PCA) is implemented in an FPGA to detect moving objects within a scene. The complete integrated development of the PCA algorithm on an FPGA was first achieved in these works. In [38,39] an FPGA is employed to implement a remote control system based on networked robot manipulators. The image processing task is divided into two principal data flows. On the one hand, the image taken by the camera located at the robot end-effector is binarized (which represents a low-level vision task) before object descriptors are obtained (a mid-level vision task). Then, FPGA network interface is employed to send the object moments using the SNRP protocol. On the other hand, the image (transformed into a grayscale image) is combined with visual information from the previous data flow. Therefore, the output image of the FPGA is an augmented reality image in which the object centroid position is marked. In order to track an object in the image using a pan-tilt camera, Perez describes an FPGA implementation of a blob's center of gravity computation in real-time [108]. These visual features feed a visual servoing scheme. Recently, a comparison made between three smart camera architectures has demonstrated that FPGA architecture is the better alternative to develop tasks like distance and angle computation of objects relative to camera [109].

Telescopes must deal with several problems related to real time image processing. Modern large telescopes require adaptive optics. Atmospheric turbulence must be on-line compensated, which requires a huge amount of processing power. To solve this, [110] summarizes the early results of a real telescope adaptive optics system based on an FPGA approach. The system has been installed in the OGS telescope at the “Observatorio del Teide” (Tenerife, Spain). This system is embedded on a Xilinx Virtex-4 FPGA. The conceptual design of an FPGA-based slope processor for the wavefront sensors of laser guide stars of extremely large telescopes is presented in [111,112]. The main concepts involved are the use of the subaperture as the finest grain for the parallel processing, the need of a different stream processor for every detector output, and the use of the row of subapertures (or equivalent subset) to determine the reuse of processing hardware. In [113], the same authors develop an FPGA phase recoverer for their CAFADIS camera. The designed phase recoverer carries out the calculations inside the atmospheric characteristic time using really high sampling. A bidimensional Fast Fourier Transform is implemented over the FPGA architecture as nuclei algorithm of the recoverer.

### 3.3. High-Level Vision Tasks

High-level vision interprets the scene through specific tasks such as relational reasoning, knowledge building, object recognition, *etc.* A task in this group is a decision task based on vision, like face-detection shown in [114]. The most important feature of an FPGA for these operations is low-power consumption. High-level tasks are decision tasks that may reduce sensor data transmission requirements. Adding high-level algorithms to a sensor is a great improvement for very remote sensor like the ones embedded on a satellite.

Hyperspectral imaging is a technique that attempts to identify features on the surface of the Earth using sensors that generally provide large amounts of data. Normally, this data is usually collected by a satellite or an airborne instrument and sent to a ground station that processes it. Thus, the bandwidth connection between the satellite and the station limits the information that can be sent and processed in real time. An on-board system that computes the great quantities of images in real-time increases the system performance [115]. Therefore, the satellite may only send the important information, and not all of the images to be processed in the ground station. The work presented in [115] integrates the Winter's N-FINDR algorithm [116] in an FPGA in order to identify the pixels defining several surfaces. In [117–119] Gonzalez *et al.* implement the Pixel Purity Index (PPI) algorithm over an FPGA to obtain these interesting points in the ground photographed by the satellite. Later, in [119,120], they develop a parallel FPGA-based design of the Image Space Reconstruction Algorithm (ISRA) to sort out the same problem of surface detection using hyperspectral image sensors.

Another high-level vision task related to the satellite photography is described in [77]. The main contribution of this paper is the design of an adviser FPGA approach capable of predicting the reconstruction error of an image when it is compressed with different techniques to a fixed compression ratio, that is, it can advise to the on-board compression system what kind of compression algorithm is more suitable for the satellite requirements. In most cases, this coprocessor will decide whether the on-board JPEG2000 compression system must apply the lossless or lossy algorithm. Sometimes, when high-level vision processing task are required, the hardware design implements a microprocessor embedded on the FPGA (e.g., Xilinx Microblaze) that could run a C-programmed algorithm and be executed without any noticeable restriction from a console application on a desktop PC. In [121] this technique is employed for an embedded vision sensor to track and count people. Sometimes, the FPGA is used in a vision system only to control the image data flow over specific DSP processors. In [122] the hardware architecture of a smart video sensor node was developed using two DSP processors and an FPGA that controls, in a flexible way, the interconnection among processors and the image data flow. The video sensor node processes images locally in order to extract objects of interest, and classify them.

## 4. FPGAs Employed in Sensor Systems

In this section, the particular characteristics of FPGA devices from several manufacturers are briefly described. The two main FPGA manufacturers in terms of market share are Xilinx and Altera, although there are several others that provide FPGAs like Actel, Atmel, Lattice Semiconductor, *etc.* The characteristics of current products from each of these are described and compared in turn. Of particular interest from a sensor systems' perspective is the power consumption primarily, besides of size of the device in terms of logic resources, embedded memories, embedded multipliers or DSP blocks, and whether or not the device includes a processor core.

### 4.1. Xilinx

Xilinx was one of the first developers of field programmable gate array technology. It has had a number of devices' families, with the two current families being the Spartan series and the Virtex series. The main difference between the two families is that the Spartan devices are designed primarily for low cost, and theVirtex devices are designed primarily for high performance. Recently, Xilinx has focused on reducing the power consumption of its devices using integrated optimized hard-core blocks, for instance the Virtex-II Pro family devices have two PowerPC 405 hard-core processors. This processors permit to virtually add any peripheral or create custom accelerators that extend system performance.

The Spartan series is employed for low-power design, cost sensitivity and high-volume; e.g., displays, wireless routers and other applications. The Spartan-6 family is built on a 45 nanometer, 9-metal layer, dual-oxide process technology. The Spartan-6 was marketed in 2009 as a low-cost solution for automotive, wireless communications, flat-panel display and video vigilance applications. Furthermore, most of sensor systems designers employ the Spartan-III or Spartan 6 FPGAs due to its low cost and low energy consumption. In [81], the Spartan 3AN-50 device has been used to implement tactile sensors taking advantage of its numerous I/O pins, compact size and low cost. In [41], the authors used the XC3S2000 device from Spartan-III family to achieve a real-time fuzzy controller. The fuzzy algorithm has been designed with the goal of developing a real-time FPGA-based controller. Therefore, the complexity has been reduced, while keeping a great degree of parallelism. Other works use the computational power of the Spartan III FPGAs to achieve vision and image processing tasks [88,121,122].

The Virtex series of FPGAs integrate features that include FIFO logic, DSP blocks, PCI-Express controllers, Ethernet MAC blocks, and high-speed transceivers. In addition to FPGA logic, the Virtex series include embedded fixed function hardware for commonly used functions such as multipliers, memories, serial transceivers and microprocessor cores. Xilinx's most recently Virtex family, the Virtex 7, is based on a 28 nm design and is designed to deliver a two-fold system performance improvement at 50% lower power compared to previous generation Virtex-6 devices. In addition, Virtex-7 doubles the memory bandwidth compared to previous generation Virtex FPGAs with 1,866 Mbit/s memory interfacing performance and over two million logic cells. Sensor systems designers in Spain tend to use devices like Virtex-E [50,55,73,74], Virtex-II [38,39,107] and Virtex-II Pro [33,106,117] to achieve complex sensing systems and specifically vision systems or wireless sensor networks because such FPGAs are a powerful high performance devices at a reasonable costs.

In the newest generation, the 7 Series devices, the Spartan family is replaced by the Artix and Kintex families. Within each generation, a range of device sizes is available. Table 1 depicts the characteristics and the static power consumption (calculated via the Xilinx Power Estimator tool XPE that is not available for old FPGA families, where it has replaced by “-” in the table) of the different FPGA series of Xilinx.

### 4.2. Altera

Currently, Altera provides three families of FPGA devices: the Cyclone series (low cost), the Arria series (mid-range) and the Stratix series (high performance). None of these families incorporate a hard-core processor within the logic but Altera has focused its efforts on its soft-core processor called NIOS processor, or NIOS-II in its newest FPGA devices (The last FPGA family from Altera that had a hard-core processor was the Excalibur FPGA family witch integrated a microprocessor subsystem called ARM922T).

The Cyclone series was designed for low cost applications, making it well suited for sensor systems including embedded image processing applications. The FPGA family most recent from cyclone series is the Cyclone VI based on 4-input LUT (Look Up Table) with a register on the output. It incorporates dedicated hardware multiplication blocks to achieve a single multiplication of 18-bit numbers or tow multiplications of 9-bit numbers, also it has a configurable-size embedded memory blocks up to 150 Kbits. The performance of the I/O blocks has been improved to support a variety of interface standards like DDR/QDR memories, PCI express and others. In [20], the EP1C6T144CSN device from Altera's Cyclone FPGA family was used to implement an intelligent Front-End Signal Conditioning Circuit for IR Sensors.

In Stratix FPGAs, The basic structure is similar to that of the Cyclone but with much more improvements, where the LUT here has 8 inputs with 28 nm process (for Stratix V devices), furthermore incorporate sophisticated DSP blocks up to 54 × 54 precision and have 20 Kbit Ram blocks that can be configured as dual-port RAM, FIFO or shift registers.

The Arria series based on 8-input LUT, integrate high speed transceiver blocks designed primarily for high performance serial communication applications. The other features of Arria FPGAs are basically the same as that of the Stratix. Table 2 depicts the characteristics and power consumption of Altera FPGA families (power consumption was calculated via PowerPlay Early Power Estimators tool (not available for Excalibur family)).

### 4.3. Other FPGA Providers

Actually, there are various companies that produce and provide FPGA devices like Actel, Lattice Semiconductor, Atmel, Tabula, SiliconBlue, Achronix, QuickLogic, MathStar, Cypress and others.

Actel provides a range of low power FPGAs, making them ideally suited for sensor systems. There are three main families: the Axcelerator, the ProASIC3 and the IGLOO. The IGLOO family is a low power, based on 3-input LUT and 130 nm process technology, its RAM is a true dual-port memory and the larger devices are able to implement a 32-bit ARMprocessor as a soft-core block. In [17], the authors implement a low-cost camera sensor based on Actel IGLOO FPGA that fits its low power consumption, reprogrammability and cost requirements.

Lattice Semiconductor produces a number of FPGA families. Its current families are the ECP series (low cost), the XP series (non-volatile) and the SC/M family (high performance). The XP FPGAs contain an on-chip no-volatile flash memory that may be used to configure the FPGA on power-up. This saves the need of an external flash memory and consequently reduces design costs.

## 5. Conclusions

To conclude, FPGA devices have reached a high level of development that puts them in competition with the traditional application specific integrated circuits (ASICs) in terms of performance, power consumption and cost. In just two decades, they have turned from merely simple Hardware-Prototyping tool into an impressive solution for those system designs that require a very high level of accuracy, powerful computational capabilities and real parallel execution. From a technological perspective, the industry of FPGA devices has made great strides from simple FPGA chips for prototyping purposes only that included a few hundred logic cells and small blocks of memory to FPGAs with the 28 nm process technology. These last FPGAs include more than two million logic cells, several types of memories and peripheral interfaces. This is the case of the Virtex-7 FPGA devices family from Xilinx and the Stratix-V from Altera.

As described throughout the paper, research on FPGA-based sensor systems in Spain is well established. The capabilities of the new FPGAs allow providing the sensor systems with different functions such as self-diagnosis, signal processing, communications in a WSN, *etc.* Currently, the term smart sensor is employed to refer these sensor systems with some kind of “intelligence”. Furthermore, we have described interesting research related with these FPGA-based sensor systems. Within this research, we can mention several works like the implementation of real time signal processing from the obtained sensory information, the use of parallel architectures to process a great quantity of information, to implement and optimize sensor-based controllers in embedded systems, the use of WSNs to process sensory information using different FPGA-based network nodes, *etc.* Computer vision systems have specially been enhanced by the use of FPGAs. From low-level vision tasks like basic point pixel operations or simple filtering image processing, through mid-level tasks that compute visual features like image moments, until high-level vision tasks where the FPGA allows taking important decisions by processing an image, camera sensors have been considerably enhanced. Different works employ FPGA in sensor systems to implement parallel algorithms in order to process data in a low-power consumption device. These algorithms use FPGA architectures not only to implement simple combinational and sequential circuits, but also to include high-level operations in embedded systems. The optimal implementation of these algorithms using the capabilities of the new FPGAs will suppose an important research field in the near future.

## Figures and Tables

**Table 1.** Characteristics, static power consumption and related works of the different FPGA series of Xilinx.

**Family**	**Process (nm)**	**LUT Size**	**Logic Cells**	**RAM (bits)**	**Number of DSP Blocks**	**HC-Processor**	**Static Power Consumption (mW)**	**Related Works**
**Spartan-II**	180	4	432–5.3 k	16 K–56 K	-	-	-	[18,34,35,42,45,87]
**Spartan-III**	90	4	1.5 k–66 k	72 k–1.8 M	4–104	-	27–336	[3,4,14,19,30,35,37,41, 51–53,56,62,65,66, 68,76,81,86,88,121–124]
**Spartan-6**	45	6	2 k–147 k	144 K–4.8 M	4–180	-	11–94	[24,67]
**Artix-7**	28	6	11 k–215 k	720 K–13 M	40–740	-	68–122	-
**Kintex-7**	28	6	19 k–477 k	2.3 M–34 M	120–1920	-	79–216	-
**Virtex**	220	4	1.7 k–27 k	32 K–128 K	-	-	-	-
**Virtex-E**	180	4	1.7 k–73 k	65 K–851 K	-	-	-	[2,25,26,32, 48–50,55,73,74]
**Virtex_II**	120/150	4	512–93 k	72 K–128 K	4–168	-	-	[38,39,102,107]
**Virtex-II Pro**	90/130	4	2.8 k–88 k	216 K–7.8 M	12–444	PowerPC 405	-	[33,78,106,117–119]
**Virtex-4**	90	4	12 k–200 k	648 K–9.7 M	32–96	PowerPC 405	128–1278	[64,86,110,112,113, 115,120]
**Virtex-5**	65	6	12 k–415 k	936 K–18 M	32–1056	PowerPC 405	276–3028	[47,65,80,87,104,105,111]
**Virtex-6**	40	6	46 k–474 k	5.5 M–37 M	288–2016	-	715–4441	[36]
**Virtex-7**	28	6	179 k–1954 k	14 M–68 M	700–3600	-	177–1250	-

**Table 2.** Characteristics and static power consumption of the different FPGA series of Altera.

**Family**	**Process (nm)**	**LUT Size**	**Logic Cells**	**RAM (bits)**	**Number of DSP Blocks**	**HC-Processor**	**Static Power Consumption (mW)**
Excalibur	180	4	4 k–34 k	32 K–256 K	-	ARM922T	-
Cyclone	130	4	2.9 k–20 k	58 K–288 K	-	-	48–120
Cyclone II	90	4	4.6 k–64 k	117 K–1.1 M	13–150	-	29–193
Cyclone III	65	4	5.2 k–119 k	414 K–3.8 M	23–288	-	55–150
Cyclone IV	60	4	6.3 k–150 k	270 K–6.3 M	15–266	-	60–152
Arria GX	90	8	8 k–36 k	1.2 M–4.3 M	10–44	-	405–826
Arria II GX	40	8	6 k–102 k	783 K–8.3 M	29–92	-	329–793
Stratix	130	4	10 k–79 k	899 K–7 M	6–22	-	187.5–1,395
Stratix II	90	8	6 k–72 k	410 K–8.9 M	12–96	-	323–1,435
Stratix III	65	8	19 k–135 k	1.8 M–14 M	27–112	-	404–1,255
Stratix IV	40	8	29 k–325 k	6.3 M–22 M	48–161	-	436–1,739
Stratix V	28	8	239 k–1,087 k	29 M–53 M	200–1,840	-	641–1,153
